# MicroRNA-29b/29c targeting CTRP6 influences porcine adipogenesis via the AKT/PKA/MAPK Signalling pathway

**DOI:** 10.1080/21623945.2021.1917811

**Published:** 2021-05-02

**Authors:** Wenjing Wu, Ke Xu, Meng Li, Jin Zhang, Yizhen Wang

**Affiliations:** a Key Laboratory of Animal Nutrition & Feed Sciences, College of Animal Sciences, Zhejiang University, Hangzhou, China; b College of Biological and Chemical Sciences and Engineering, Jiaxing University, Jiaxing, China

**Keywords:** miR-29b/c, porcine adipocytes, gene expression, CTRP6, adipogenesis

## Abstract

Porcine fat deposition is an important economic trait of pig breeds, and understanding the gene regulatory network in adipocytes is essential for modern pig breeding. In a previous study, we demonstrated that miR-29a negatively regulates the differentiation of porcine adipocytes. In this study, we investigated the roles of miR-29b/c in porcine adipocytes and the underlying mechanisms. Using EdU staining and the CCK-8 assay, we observed that transfection with the miR-29b/c agomir promoted the proliferation of porcine intramuscular (IM) and subcutaneous (SC) adipocytes by altering the expression of cell-cycle-related genes. According to the results of oil red O staining and western blot analysis, transfection with the miR-29b/c agomir suppressed the differentiation of porcine SC and IM adipocytes via the AKT/PKA/MAPK signalling pathway. Furthermore, we proved that miR-29b/c regulates porcine adipocytes by directly targeting the 3ʹ-untranslated region (3ʹUTR) of CTRP6 using a dual-luciferase reporter assay. Finally, co-transfection with miR-29b/c and CTRP6 partially restored the changes of phenotype and gene expression induced by miR-29b/c overexpression in 3T3-L1 adipocytes. Taken together, our data demonstrate that both miR-29b and miR-29 c negatively regulate porcine adipogenesis by targeting CTRP6, which furthers our understanding of the gene network that regulates fat deposition in pigs.

## Graphical Abstract

**Figure:**
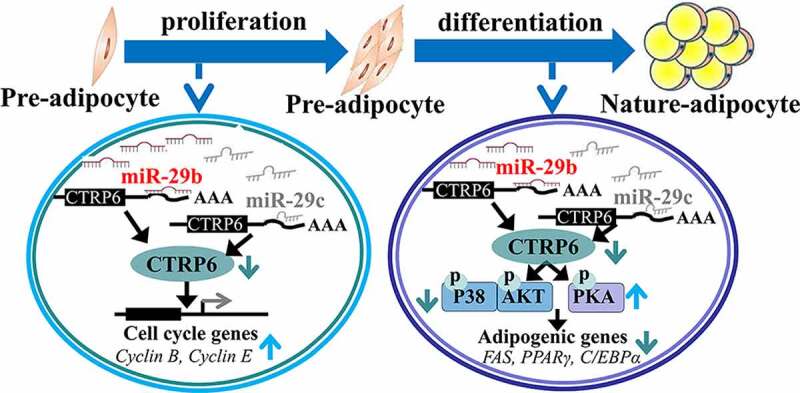

## Introduction

Porcine fat deposition is one of the most important economic traits in pig husbandry. The intramuscular (IM) fat content is the key factor of pork quality, and the subcutaneous (SC) fat amount is negatively associated with the lean percentage of the carcase [1]. In the past decades, selection oriented on carcase lean-meat percentage has led to a dramatic decrease of SC fat deposition, which improved pork production efficiency [2]. However, the IM fat content also decreased under the selection, which resulted in mediocre pork quality. Thus, one major goal of modern pig breeding is to improve the IM fat content without increasing the amount of SC fat, resulting in high IM/SC ratios [3]. Understanding the gene regulatory network and elucidating the mechanisms of regional fat distribution is helpful for breeding pigs with high IM/SC ratios.

MicroRNAs (miRNAs) are non-coding RNAs with a length of 19–22 nt that can bind to the 3ʹ untranslated region (UTR) of a targeted mRNA, leading to its digestion or impeding the transcription of target genes, thereby affecting a variety of cellular behaviours [4]. A growing body of research indicate that miRNAs play vital roles in the proliferation and differentiation of adipocytes [5]. The miR-29 family comprises three mature members, miR-29a, miR-29b and miR-29 c, which are encoded by two gene clusters. These miRNAs are highly expressed in insulin-sensitive tissues and are upregulated in rodent models of obesity or diabetes [6]. A recent meta-analysis of miRNA expression profiles of patients with type 2 diabetes or rodent models of diabetes identified miR-29a as the most upregulated miRNA across different insulin-sensitive tissues [7]. Overexpression of miR-29a in adipocytes inhibits insulin-stimulated glucose uptake, and was also found to negatively regulate gluconeogenesis and insulin signalling in hepatocytes [8]. Fatty acid oxidation is negatively regulated by miR-29a/c overexpression, potentially by regulating the expression of peroxisome proliferator-activated receptor γ coactivator-1α [9]. In our previous research, we found that miR-29a negatively regulates the differentiation of SC and IM adipocytes of pigs by targeting complement-C1q/tumour necrosis factor-related protein 6 (*CTRP6*), a gene that is known to promote adipocyte adipogenesis [10]. However, the functions of miR-29b and miR-29 c and their target genes remain unknown.

In this study, we investigated the roles of miR-29b/c in porcine SC and IM adipocytes, as well as the underlying cellular mechanisms. The results indicated that miR-29b/c accelerated the proliferation and inhibited the differentiation of SC and IM adipocytes by regulating genes related to the cell cycle and adipogenesis, respectively. We also demonstrate that *CTRP6* is a target gene of miR-29b/c, through which it regulates the proliferation and differentiation of porcine adipocytes via the MAPK/AKT/PKA pathway. Our findings provide new knowledge on the roles of miR-29 in porcine adipocytes and further our understanding of the gene network that regulates fat deposition in pigs.

## Material and methods

### Animals

Three 3-day-old piglets of Jiaxing (JX) black pig were provided by Zhejiang Qinglian Food Co., Ltd (Jiaxing, Zhejiang Province, China). The longissimus thoracis muscle and subcutaneous adipose tissues were collected from the piglets after they were euthanized with sodium pentobarbital. The animal care was in accordance with the guidelines of the Jiaxing University Animal Care Committee.

### Cell culture and adipocyte differentiation

The longissimus thoracis muscle and subcutaneous adipose tissue were collected from the pigs under aseptic conditions. The isolated tissue samples were macerated and digested with 1 mg/mL collagenase type I (Invitrogen, Carlsbad, CA, USA) at 37°C for 60 min, then filtered through a 75 μm nylon mesh. Adipose-derived stromal-vascular (SV) cells were collected by centrifugation at 1360 × *g* for 10 min and grown in DMEM/F12 containing 10% foetal bovine serum (FBS, Gibco, USA) and 1% antibiotic/antimycotic solution (15,140–122, Gibco, USA) in a humidified atmosphere with 5% CO_2_ at 37°C. The cells were cultured to confluence (day 0) in growth medium, and then differentiated for 2 days in differentiation medium (DMEM/F12 with 10% FBS, 20 nM insulin, 0.5 mM dexamethasone, 0.5 mM isobutyl methylxanthine (IBMX). The cells were then maintained in DMEM/F12 containing 10% FBS and 20 nM insulin for another 4–6 days. During differentiation, the medium was replaced every other day [11].

### EdU detection

For this assay, 10 μM 5-ethynyl-2ʹ-deoxyuridine (EdU, RiboBio, Guangzhou, Guangdong, China) was added into the growth medium and incubated for 3 h. Fixation, permeabilization, and EdU staining were done according to the manufacturer’s protocol. Cell nuclei were counter-stained with Hoechst 33,342 (Invitrogen, Carlsbad, CA, USA) at a concentration of 5 μg/ml for 10 min. Then, EdU-positive cells were visualized under a fluorescence microscope (Nikon, Tokyo, Japan) to calculate the ratio of EdU-positive cells (EdU-stained cells/total cells) [10].

### CCK-8 detection

Adipocytes were seeded into 96-well plates at 5 × 10^3^ cells per well in 100 μl of growth medium. At 48 h after treatment with miR-29b agomir or miR-29 c agomir, the CCK-8 kit (Beyotime, Shanghai, China) was used to detect cell proliferation according to the manufacturer’s instructions [10].

### Oil Red O staining

The miR-29b agomir or miR-29 c agomir treated cells were matured for 8 days, then washed with PBS, fixed with 4% paraformaldehyde for 30 min at room temperature, and washed again three times with PBS. The fixed cells were then covered with a mixture of Oil Red O solution (0.6% Oil Red O dye in isopropanol) and water at a 6:4 ratio for 30 min, followed by washing four times with PBS, and images were captured under an optical microscope (Nikon, Tokyo, Japan) [12].

### Triglyceride content assay

On day 8 of differentiation after transfection with agomir, the intracellular triglyceride content was measured using a commercial triglyceride assay kit (Nan Jing Jian Cheng Bioengineering Institute, China) according to the manufacturer’s instructions [13].

### Quantitative real-time PCR

RNA was isolated using Trizol reagent (Invitrogen, Carlsbad, CA, USA) and reverse-transcribed into cDNA using random primers and the M-MLV enzyme (Invitrogen, Carlsbad, CA, USA). Quantitative real-time PCR was performed using specific primers (**Supplementary Table 1**) and SYBR Green master mix on a BioRad iQ5 system (Bio-Rad, Hercules, California, USA). Each sample was run in triplicate. The relative mRNA abundance of each gene was normalized to the expression level of the housekeeping gene β-actin [14].

### Western blot analysis

RIPA buffer (Beyotime, Shanghai, China) supplemented with protease inhibitor (Pierce, Bradenton, Florida, USA) was used to collect the total protein. The lysates were centrifuged at 5000rpm for 30 min, and the supernatant was boiled in sodium dodecyl sulphate (SDS) loading buffer (Beyotime, Shanghai, China). After separation on a 12% polyacrylamide SDS-PAGE gel, the protein bands were transferred onto a polyvinylidene difluoride membrane (CST, Danvers, Massachusetts, USA). The membrane was then blocked in 5% defatted milk and incubated at 4°C overnight with primary antibodies followed by horseradish peroxidase-conjugated secondary antibodies. The primary antibodies included antibodies against the aP2 (ab23693, abcam, Cambridgeshire, England, Britain), FAS (ab22759, abcam, Cambridgeshire, England, Britain), HSL (ab45422, abcam, Cambridgeshire, England, Britain), phosphorylated-HSL Ser853 (ab109400, abcam, Cambridgeshire, England, Britain), ATGL (ab99532, abcam, Cambridgeshire, England, Britain), phosphorylated-AKT Ser473 (#9271, Cell Signalling Technology, Danvers, MA, USA), AKT (#9272, Cell Signalling Technology, Danvers, MA, USA), phosphorylated-PKA Thr197 (#4781, Cell Signalling Technology, Danvers, MA, USA), PKA (#4782, Cell Signalling Technology, Danvers, MA, USA), phosphorylated-p38 Thr180/Thr182 (#9211, Cell Signalling Technology, Danvers, MA, USA), p38 (#9212, Cell Signalling Technology, Danvers, MA, USA) and β-tubulin (KM9003T, Sungene Biotech, Tianjin, China). Protein bands were visualized using chemiluminescence reagents (Millipore, Massachusetts, USA) and analysed using Quantity One 4.6.3 Image [15].

### Statistical analysis

All data were derived from at least three independent experiments and presented as means ± SEM. Differences between groups were analysed using Student’s two-tailed *t*-test when only two groups were compared, or using single-factor analysis of variance (one-way ANOVA) when more than two groups were compared. Differences with *P*-values <0.05 were considered statistically significant.

## Results

### Expression profiles of miR-29b/c in porcine tissues and during adipocyte differentiation

**Figure 1.**
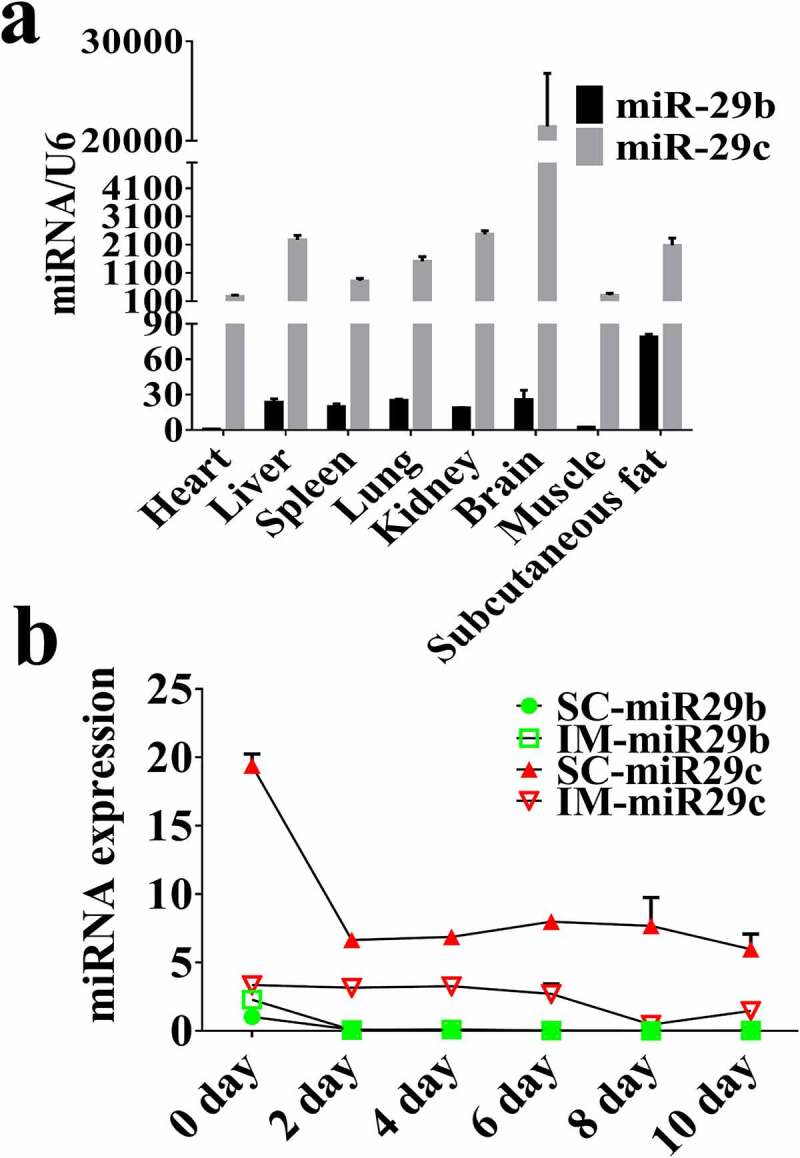
**miR-29b/c expression in various tissues and during adipocyte differentiation** (a) Expression of miR-29b/c in various porcine tissues. (b) Expression of miR-29b/c during the differentiation of intramuscular and subcutaneous adipocytes

After searching the miRbase database (Release 22.1) for the mature mammalian sequences of miR-29b/c, we noticed that the seed region of miR-29b/c was 100% conserved among many mammals **(Supplementary Figure 1)**, which indicated an important function that did not change through evolution. To obtain clues on the potential functions of miR-29b/c, we analysed their expression profiles in porcine tissues by qPCR. Both molecules were detected in the seven investigated tissues, but the expression level of miR-29 c was over 10-fold higher than that of miR-29b in all tissues (Figure 1(a)). SC fat exhibited the highest level of miR-29b and second highest level of miR-29 c. Based on this finding, we next analysed the changes of miR-29b/c expression during the differentiation of porcine SC and IM adipocytes *in vitro*. A significant downregulation of miR-29b/c was observed in both types of adipocytes (Figure 1(b)). These results indicated that miR-29b/c were both negatively associated with adipogenesis in porcine adipocytes.

### MiR-29b/c promote the proliferation of porcine adipocytes

**Figure 2.**
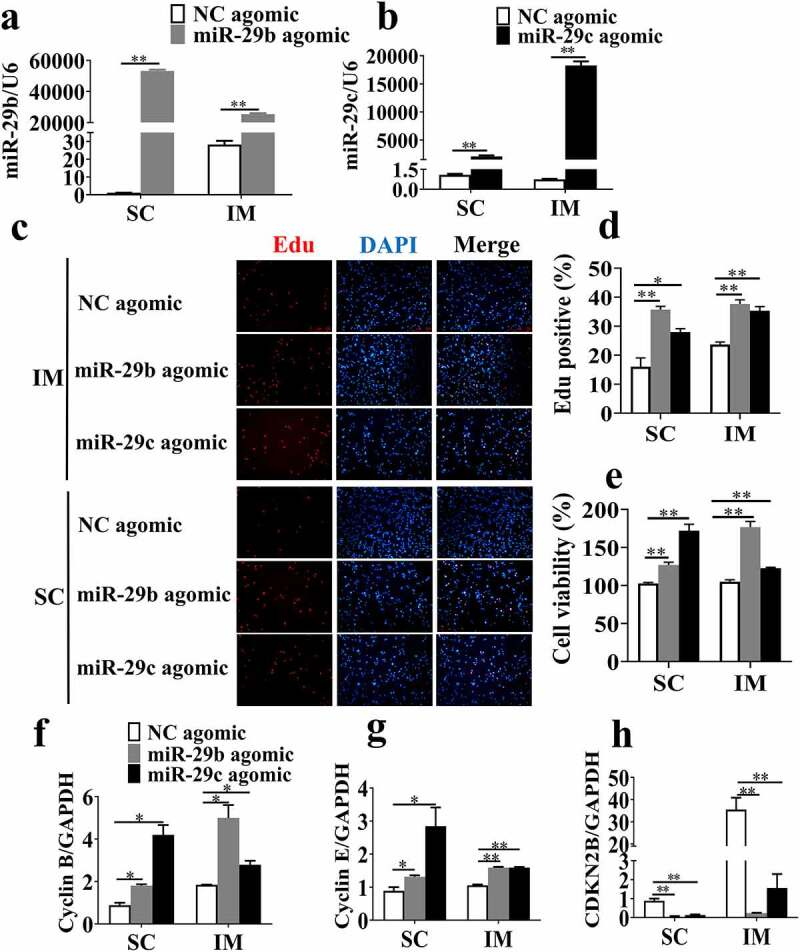
**Overexpression of miR-29b/c promotes the proliferation of porcine adipocytes**. Porcine adipocytes were transfected with miR-29b/c agomir for 24 h (a-b). (c) The proliferation of intramuscular and subcutaneous adipocytes was examined using the EdU assay. Red represents EdU staining, and blue represents cell nuclei conter-stained with Hoechst 33,342. (d) The percentage of EdU-positive cells was quantified. (e) Cell proliferation was examined using a CCK-8 kit. (f-h) The expression levels of cyclin B, cyclin E and CDKN2B were determined by real-time quantitative PCR and normalized to the GAPDH level. The data represent means ± SEM. n = 3, *P < 0.05, **P < 0.01

To test their cellular function, primary porcine SC and IM adipocytes were transfected with miR-29b/c agomir or NC agomir. The expression of miR-29b/c was over 1000-fold higher in the miR-29b/c agomir groups than in the NC agomir groups, which indicated that the expression efficiency of miR-29b/c agomir was sufficient for further analysis (Figure 2(a-b)). To analyse the effects of miR-29b/c on adipocyte proliferation, EdU staining (Figure 2(c-d)) and CCK-8 assay (Figure 2(e)) were performed. Both assays indicated that the cell proliferation of both SC and IM adipocytes was enhanced by transfection with miR-29b/c agomir. Furthermore, cell cycle related genes (cyclin B and cyclin E) were upregulated (Figure 2(f-g)) and a cell cycle repressor gene (CDKN2B) was downregulated in the miR-29b/c agomir group (Figure 2(h)). Taken together, these data demonstrated that miR-29b/c could promote the proliferation of porcine SC and IM adipocytes.

### MiR-29b/c suppress porcine adipocyte differentiation

**Figure 3.**
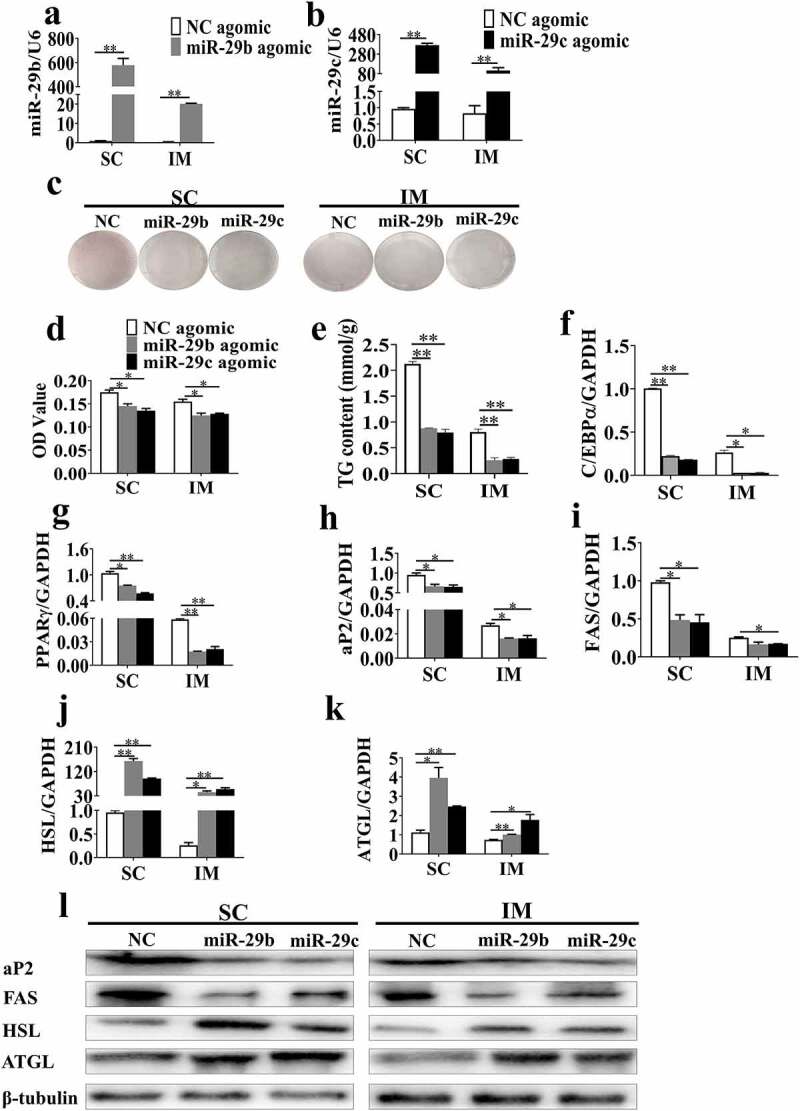
**Overexpression of miR-29b/c inhibits lipid accumulation in adipocytes**. After transfection with miR-29b/c agomir and induction of adipogenic differentiation, the overexpression efficiency of miRNA was confirmed (a-b). (c) subcutaneous (SC) and intramuscular (IM) adipocytes were stained with oil red O at day 10. The intracellular lipid content was determined by oil red O staining in SC and IM adipocytes (d) on day 10, as well as the triglyceride content in SC and IM adipocytes (e). (f-k) mRNA levels of *C/EBPα, PPARγ, FAS, aP2, HSL* and *ATGL* according to real time qPCR analysis. (l) Protein levels of adipogenic markers after transfection and induction of differentiation for 10 days. The data represent means ± SEM. n = 3, *P < 0.05, **P < 0.01

After miR-29b/c agomir transfection and adipogenic induction for 8 days, the expression levels of miR-29b/c were significantly higher in the transfected group than in the control group (Figure 3(a-b)). Oil Red O staining (Figure 3(c)) and extraction (Figure 3(d)) analysis showed that overexpression of miR-29b/c resulted in a decrease of intracellular triglyceride (TG) content in SC and IM adipocytes at day 8. The TG content assay demonstrated that SC and IM adipocytes respectively exhibited a 58.6 and 67.7% decrease of the TG content in the miR-29b agomir group, as well as 62.6 and 64.9% in the miR-29 c agomir group, compared with the NC agomir group (Figure 3(e)). Furthermore, the expression of adipogenesis marker genes (*C/EBPα, PPARγ, aP2* and *FAS*) was downregulated, while lipolytic marker genes (*HSL* and *ATGL*) were upregulated in SC and IM adipocytes transfected with the miR-29b/c agomir (Figure 3(f-k)). Overexpression of miR-29b/c also visibly decreased the protein levels of FAS and aP2 while increasing those of p-HSL, HSL and ATGL in SC and IM adipocytes (Figure 3(l)**, Supplementary Figure 2**). Therefore, these data clearly showed that miR-29b/c suppressed the differentiation of porcine SC and IM adipocytes.

### MiR-29b/c regulate adipogenic differentiation via the AKT/PKA/MAPK signalling pathway

**Figure 4.**
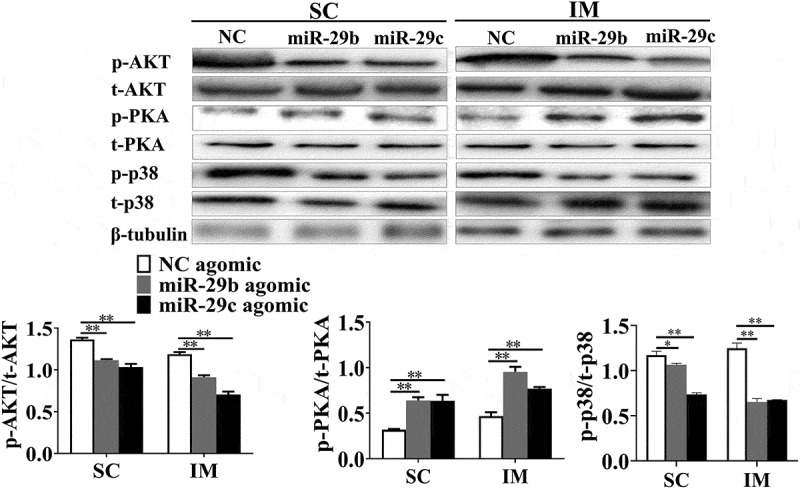
miR-29b/c regulates adipogenesis via AKT/PKA/MAPK signalling in porcine adipocytes

The AKT/PKA/MAPK signalling pathway plays a pivotal role in the proliferation and differentiation of adipocytes. To investigate whether miR-29b/c regulate adipogenesis through the AKT/PKA/MAPK signalling, the phosphorylation levels of AKT, PKA and p38 were investigated by western blot analysis. As shown in Figure 4, overexpression of miR-29b/c decreased the phosphorylation of AKT and p38, but increased the phosphorylation of PKA in both SC and IM adipocytes. This suggests that miR-29b/c inhibit AKT and p38, while activating the PKA signal transduction pathway, which might explain the decreased adipogenesis in porcine adipocytes.

The protein levels and phosphorylated status of AKT/PKA/MAPK signal transduction proteins were investigated by western blot analysis after transfection and induction of differentiation for 10 days. Quantification was performed densitometrically using ImageJ and the data re expressed as means ± SEM. n = 3, *P < 0.05, **P < 0.01.

### CTRP6 is a direct target of miR-29b/c

**Figure 5.**
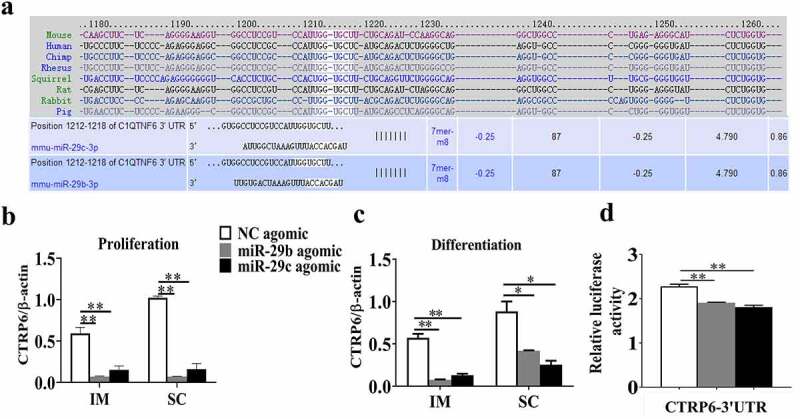
*CTRP6* is a direct target of miR-29b/c in porcine adipocytes

To further reveal the underlying mechanism through which miR-29b/c affect the proliferation and differentiation of SC and IM adipocytes, target gene prediction was conducted using the online databases TargetScan and miRbase, which predicted *CTRP6* as a candidate target gene for both miR-29b and miR-29 c (Figure 5(a)). Then, expression levels of *CTRP6* during the proliferation and differentiation of SC and IM adipocytes were examined and a decrease was found following transfection with miR-29b/c (Figure 5(b-c)). To verify whether miR-29b/c directly targets CTRP6, we cloned its 3ʹ UTR into the psi-CHECK-2 vector next to the *Renilla* luciferase coding sequence. The results of the dual-luciferase reporter assay demonstrated that miR-29b/c interacted with the target region of *CTRP6* and dramatically decreased the expression of *Renilla* luciferase (Figure 5(d)), which indicated that *CTRP6* is indeed a direct target gene of miR-29b/c.

A schematic of the target site of miR-29b/c within the 3ʹUTR of the CTRP6 mRNA identified using TargetScan (a). (b-c) The expression of CTRP6 was detected during adipocyte proliferation and differentiation. (d) HEK293T cells were transfected with luciferase reporter vector containing the miR-29b/c target sites from the 3ʹ UTR of porcine CTRP6 and miR-29b/c agomir or negative control miRNA. Following 36 h after transfection, the dual-luciferase assay was performed. The data represent means ± SEM n = 3, *P < 0.05, **P < 0.01.

**Figure 6.**
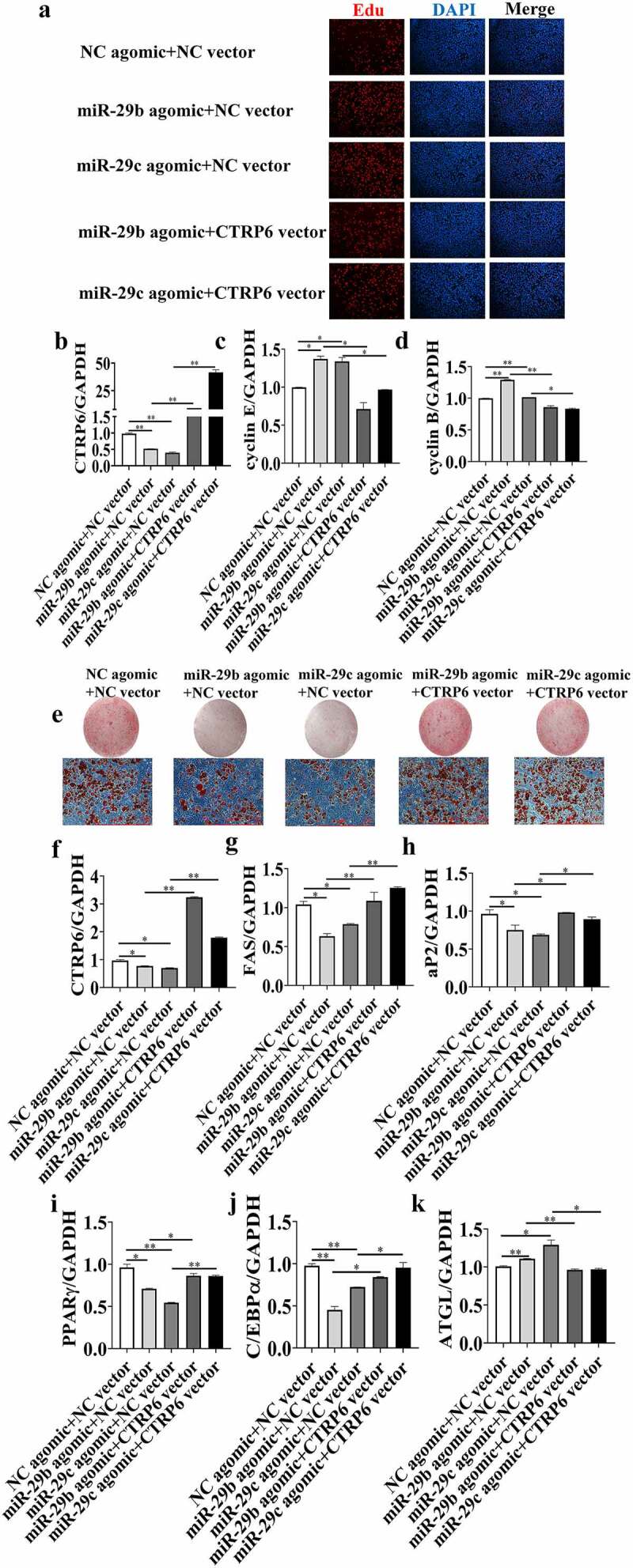
***CTRP6* overexpression attenuated the effect of miR-29b/c agomir**. Subcutaneous (SC) and intramuscular (IM) adipocytes were co-transfected with miR-29b/c agomir and s *CTRP6* expression vector. (a) The proliferation of SC and IM adipocytes was examined using the EdU assay. (b-d) Real-time qPCR analysis of *CTRP6*, cyclin B and cyclin E. At 24 h before induction of adipogenic differentiation, adipocytes were transfected with miR-29a agomir, miR-29b/c agomir and *CTRP6* vector. (e) SC and IM formation was detected with oil red O staining. Real-time qPCR analysis of the mRNA expression of adipogenic marker genes: (f) *CTRP6*, (g) *FAS*, (h) *aP2*, (i) *PPARγ*, (j) *C/EBPα*, (k) *ATGL*. The data represent means ± SEM. n = 3, *P < 0.05, **P < 0.01

To confirm the relationship between miR-29b/c and CTRP6 during adipocyte adipogenesis, 3T3-L1 cells were co-transfected with miR-29b/c agomir and the pcDNA3.1_CTRP6 vector. After 48 hours post-transfection, the cells were analysed by EdU immunofluorescence staining. The percentage of EdU-positive cells was higher in the miR-29b/c agomir group than in the NC agomir group, which was consistent with the results shown in Fig C. However, a dramatic decrease in the number of EdU-positive cells was observed in the two groups transfected with pcDNA3.1_CTRP6 (Figure 6(a)). In these two groups, CTRP6 was upregulated (Figure 6(b)) and the expression levels of Cyclin E and Cyclin B were restored (Figure 6(c-d)), which indicated that *CTRP6* overexpression was able to attenuate the function of miR-29b/c agomir.

On day 8 of adipogenic induction, Oil Red O staining (Figure 6(e)) showed that *CTRP6* overexpression recovered the TG content of adipocytes, which was inhibited by the transfection with miR-29b/c agomir. Consistent with these observations, *CTRP6* overexpression abolished the effects of miR-29b/c agomir on the expression of *C/EBPα, PPARγ, aP2, FAS* and *ATGL* (Figure 6(g-k)). These data provide further evidence that *CTRP6* is a direct and functional target of miR-29b/c in adipocytes.

## Discussion

MiRNAs are small noncoding RNAs that regulate gene expression at the post-transcriptional level, and the major function of miRNAs in adipose tissue is to promote or suppress the differentiation of adipocytes [16]. In the past decade, knowledge on the physiological roles of miRNAs in porcine adipocytes increased significantly. Many important miRNAs, such as miR-15a/b [17], miR-106a [18], miR-125a-5p [19], and miR-146a-5p [20], were identified as regulators of adipogenesis in pigs. In our experiments, we observed that miR-29b/c could promote the proliferation and suppress the differentiation of porcine SC and IM adipocytes. The underlying mechanism was explored, and the results indicated that cell cycle genes and the AKT/PKA/MAPK signalling pathway were involved, respectively. Finally, *CTRP6* was confirmed as a target gene of miR-29b/c using a dual-luciferase reporter assay and co-transfection rescue experiments. Based on our finding, miR-29b and miR-29 c have a similar function in both SC and IM adipocytes. However, the difference of their expression levels and time points during adipogenesis endow them with specific physiological roles.

*CTRP6* was identified as a direct target gene of both miR-29b and miR-29 c. CTRP6 belongs to the C1q/tumour necrosis factor-related protein family and was first reported in 2004 [21]. The expression of C*TRP6* was found to be elevated in serum and fat tissues of obese mice, ob/ob mice and adiponectin null-mice [22]. Additionally, the expression of *CTRP6* was reported to be downregulated by rosiglitazone [23]. Knockdown of *CTRP6* promotes brown adipogenesis, insulin sensitivity and attenuates diet-induced obesity via the p38MAPK/Hh signalling pathway in conjunction with the upregulation of brown fat markers and mitochondrial metabolic factors [13]. Further, we previously showed that decreasing *CTRP6* expression and secretion by shRNA knockdown promoted the proliferation and inhibited the differentiation of porcine SC and IM adipocytes [10]. In the present study, the mRNA and protein expression levels of CTRP6 decreased after overexpression of miR-29b/c in porcine adipocytes. The luciferase reporter assay demonstrated that the 3ʹ UTR of *CTRP6* contains elements interacting with miR-29b/c., which suggests that *CTRP6* is a key regulatory target of miR-29b/c in porcine adipocytes.

To further confirm these findings, cells were co-transfected with miR-29b/c and pcDNA3.1-CTRP6. Following 24 hours after treatment, pcDNA3.1-CTRP6 transfection rescued the phenotypes induced by miR-29b/c, including the expression levels of cell cycle genes (Cyclin B, Cyclin E) and the percentage of EdU-positive cells in the proliferation stage, as well as the expression levels of adipogenesis-related genes (*C/EBPα, PPARγ, FAS, ap2* and *ATGL*) and intracellular TG content in the differentiation stage. Cyclin E is the limiting factor of G1/S transition in eukaryocytes [24]. C/EBPα and PPARγ are critical transcription factors for the differentiation of adipocytes [25]. Expression changes of these genes among the five groups transfected with different constructs demonstrated that miR-29b/c regulates porcine adipogenesis via *CTRP6* as the direct target gene.

In addition to the serial induction of transcriptional regulators, modulation of intracellular signalling molecules is essential for adipocyte differentiation. It is widely accepted that insulin-induced adipocyte differentiation, adipogenesis, and TG accumulation in adipocytes involve the activation of PKA, MAPKs (ERK/p38/JNK) and Akt/PPARγ [26–28]. In the present study, miR-29b/c overexpression enhanced the phosphorylation of PKA and increased the expression of HSL. A previous study also confirmed that lipolytic mechanisms involve PKA- and PKG-dependent pathways, associated with subsequent fatty acid release via the activation of HSL [29]. This suggests that miR-29b/c may enhance lipolysis by activating the PKA signalling pathway, thereby affecting the accumulation of lipid droplets in adipocytes. We also observed that miR-29b/c reduced the activation of AKT and p38. Several studies reported that activation of AKT could induce the differentiation of 3T3-L1 adipocytes and that AKT phosphorylation was inhibited by adipogenesis inhibitors. The p38 MAPK was also found to be involved in the regulation of lipid formation in most studies describing a positive regulatory role of p38 MAPK during adipogenesis. For example, Engelman et al. showed that addition of p38 inhibitors early in 3T3-L1 differentiation decreased adipocyte formation [30]. Consistent with our previous studies, our findings indicate that decreased p38 phosphorylation and *CTRP6* expression after treatment with miR-29b/c agomir inhibited the differentiation of both types of porcine adipocytes. Therefore, our results indicate that the inhibition of adipogenesis by miR-29b/c, acting via the target gene *CTRP6*, proceeds through the AKT/p38MAPK signalling pathways.

Therefore, our study revealed that miR-29b/c are novel regulators of porcine adipocytes, which promote the proliferation and suppress the differentiation of SC and IM adipocytes by altering AKT/PKA/p38MAPK signalling pathway and targeting *CTRP6*. These findings offer new clues on the miRNA-mediated regulation of adipogenesis in porcine adipocytes.

## Supplementary Material

Supplemental Material
