# Supplementary material for: MicroRNA-29b/29c targeting CTRP6 influences porcine adipogenesis via the AKT/PKA/MAPK Signalling pathway
Source: Adipocyte. 2021 May 2;10(1):264–74. doi: 10.1080/21623945.2021.1917811 (PMC8096332; doi:10.1080/21623945.2021.1917811)
Supplement: Supplemental Material [file KADI_A_1917811_SM2707.zip › SUP.docx]

**Supplementary Table 1 Primers (S, sense; A, antisense) for real time PCR.**

| Gene | Accession  Number | Primer Sequences | Production length  /bp | Tm/℃ |
| --- | --- | --- | --- | --- |
| CTRP6 | NM_001142830.1 | S: GCTCCTGTTCCCTGTCTCCAC | 108 | 60 |
|  |  | A: TGGGGGTCACAGCATCGTC |  |  |
| PPARγ | NM_214379 | S: AGGACTACCAAAGTGCCATCAAA | 142 | 60 |
|  |  | A: GAGGCTTTATCCCCACAGACAC |  |  |
| CDKN2B | NM_214157 | S: AGTGGCGGCGGTGGAGAT | 217 | 60 |
|  |  | A: GGGTGAGGGTGGCAGGGT |  |  |
| aP2 | NM_001002817.1 | S: GAGCACCATAACCTTAGATGGA | 121 | 60 |
|  |  | A: AAATTCTGGTAGCCGTGACA |  |  |
| Cyclin B | NM_001170768.1 | S: AATCCCTTCTTGTGGTTA | 104 | 60 |
|  |  | A: CTTAGATGTGGCATACTTG |  |  |
| Cyclin E | NM_001243931.1 | S: AATCCCTTCTTGTGGTTA | 306 | 60 |
|  |  | A: CTTAGATGTGGCATACTTG |  |  |
| C/EBPα | NM_001199889.1 | S: CGCAGGTCAAGAGTAAGACCA | 192 | 60 |
|  |  | A:ACAGCTGCTCCACCTTCTTC |  |  |
| HSL | NM_214315 | S: CACTGACTGCTGACCCCAAG | 217 | 60 |
|  |  | A: TCCTCACTGTCCTGTCCTTCAC |  |  |
| FAS | EF589048.1 | S: AGCCTAACTCCTCGCTGCAAT | 196 | 60 |
|  |  | A: TCCTTGGAACCGTCTGTGTTC |  |  |
| GAPDH | DQ452569.1 | S: AGGTCGGAGTGAACGGATTTG | 118 | 60 |
|  |  | A: ACCATGTAGTGGAGGTCAATGAAG |  |  |
| ATGL | EF583921.1 | S: TCACCAACACCAGCATCCA | 95 | 62 |
|  |  | A: GCACATCTCTCGAAGCACCA |  |  |

**Supplementary Figure 1.** Comparation of miR-29b/c mature sequence from pig, human, mice, rat, cattle and horse.

**Supplementary Figure 2.**  Protein levels of p-HSL after transfection and induction of differentiation for 10 days.
