# Supplementary figures and images for: MicroRNA-29b/29c targeting CTRP6 influences porcine adipogenesis via the AKT/PKA/MAPK Signalling pathway
Source: Adipocyte. 2021 May 2;10(1):264–74. doi: 10.1080/21623945.2021.1917811 (PMC8096332; doi:10.1080/21623945.2021.1917811)

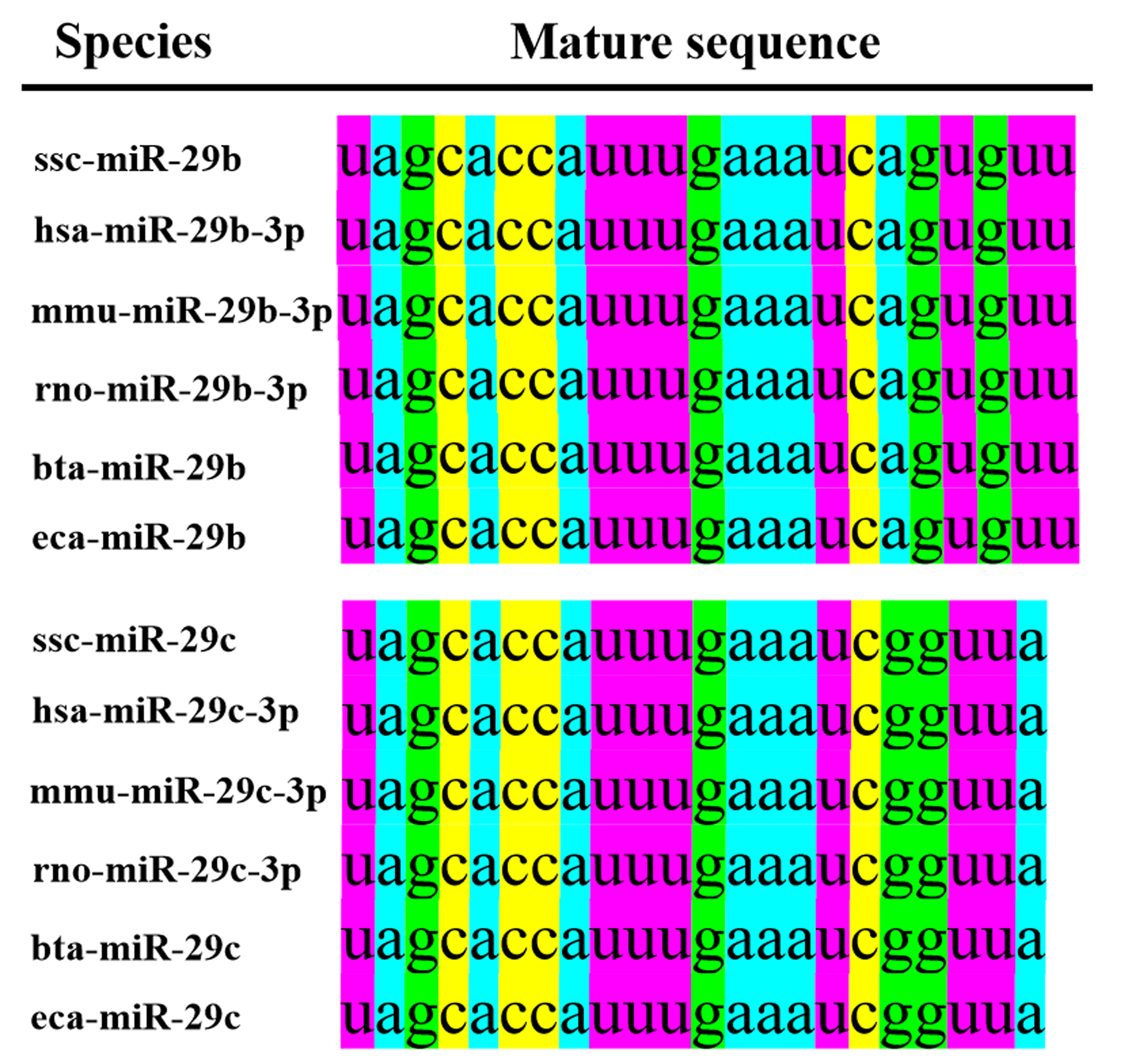

Supplement: Supplemental Material [file KADI_A_1917811_SM2707.zip › Figure S1.tif]

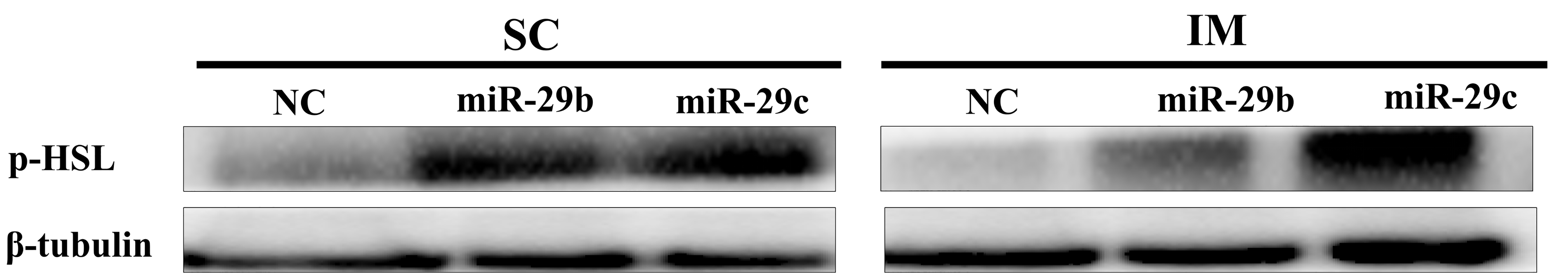

Supplement: Supplemental Material [file KADI_A_1917811_SM2707.zip › Figure S2.tif]
